# Commentary: Using QbTest for monitoring pharmacological treatment response in ADHD – are we there yet?

**DOI:** 10.1111/jcpp.14071

**Published:** 2024-11-08

**Authors:** Alessio Bellato, Valeria Parlatini, Madeleine J. Groom, Charlotte L. Hall, Chris Hollis, Emily Simonoff, Anita Thapar, Samuele Cortese

**Affiliations:** ^1^ School of Psychology University of Southampton Southampton UK; ^2^ Centre for Innovation in Mental Health, School of Psychology, Faculty of Environmental and Life Sciences University of Southampton Southampton UK; ^3^ Institute for Life Sciences University of Southampton Southampton UK; ^4^ School of Psychology University of Nottingham Malaysia Semenyih Malaysia; ^5^ Mind & Neurodevelopment (MiND) Research Cluster University of Nottingham Malaysia Semenyih Malaysia; ^6^ Solent NHS Trust Southampton UK; ^7^ NIHR MindTech HealthTech Research Centre, Institute of Mental Health, School of Medicine University of Nottingham Nottingham UK; ^8^ NIHR Nottingham Biomedical Research Centre, Institute of Mental Health University of Nottingham Nottingham UK; ^9^ Mental Health and Clinical Neurosciences, School of Medicine University of Nottingham Nottingham UK; ^10^ Department of Child and Adolescent Psychiatry, King's College London Institute of Psychiatry, Psychology and Neuroscience London UK; ^11^ Division of Psychological Medicine and Clinical Neurosciences, Wolfson Centre for Young People's Mental Health Cardiff University School of Medicine Cardiff UK; ^12^ Clinical and Experimental Sciences (CNS and Psychiatry), Faculty of Medicine University of Southampton Southampton UK; ^13^ Hassenfeld Children's Hospital at NYU Langone New York University Child Study Center New York NY USA; ^14^ DiMePRe‐J‐Department of Precision and Rigenerative Medicine‐Jonic Area University of Bari "Aldo Moro" Bari Italy

**Keywords:** ADHD, activity level, biomarkers, continuous performance test, outcome, pharmacotherapy

## Abstract

Individuals with attention‐deficit/hyperactivity disorder (ADHD) exhibit varied responses to pharmacological treatments (e.g. stimulants and non‐stimulants). Accurately and promptly detecting treatment‐related improvements, response failure, or deterioration poses significant challenges, as current monitoring primarily relies on subjective ratings. In this commentary, we critically evaluate the evidence supporting the use of QbTest for objectively monitoring ADHD treatment response in clinical practice. We also offer recommendations for future research, advocating for rigorous clinical trials and longitudinal studies to further explore the potential utilisation of QbTest and other tools for monitoring treatment responses in individuals with ADHD.

Attention‐deficit/hyperactivity disorder (ADHD) is characterised by developmentally inappropriate inattention and/or hyperactivity/impulsivity (American Psychiatric Association, 2022), which often persist into adulthood (Sibley, Mitchell, & Becker, 2016) and might lead to impairment in social, educational and/or occupational functioning, especially if not effectively treated (Faraone et al., 2021; Sayal, Prasad, Daley, Ford, & Coghill, 2018). There are several evidence‐based pharmacological interventions for ADHD, of which first‐line choices are typically stimulants (methylphenidate and amphetamines) and second‐line treatments include non‐stimulants (e.g. atomoxetine or guanfacine) (National Institute for Health and Care Excellence, 2018). Non‐pharmacological interventions include cognitive and/or behavioural therapy (e.g. parent training) (Faraone et al., 2021; Groom & Cortese, 2022). Although clinical trials have consistently demonstrated benefits of ADHD medication on core symptoms (Cortese et al., 2018) and quality of life (Bellato, Perrott, et al., 2024), there is heterogeneity in treatment response at the individual level (Coghill et al., 2023; Salazar de Pablo et al., 2024). Clinical trials have not identified any characteristics consistently associated with this variability. However, it has been suggested that clinical and demographic factors (such as age, ADHD presentation, co‐occurring physical and mental health conditions) and treatment‐related characteristics (including dose, adherence and formulations) may contribute to such heterogeneity (Hodgkins, Dittmann, Sorooshian, & Banaschewski, 2013; Ramsay, 2017).

Clinical guidelines recommend clinicians should monitor benefits (how well the current treatment is working for a specific individual), potential adverse effects (both related to physical or mental health) and treatment adherence (CADDRA – Canadian ADHD Resource Alliance, 2020; National Institute for Health and Care Excellence, 2018). Being able to accurately monitor the individual response to pharmacological treatment for ADHD is therefore crucial for clinicians to make recommendations regarding dose or medication changes, especially during treatment initiation, to optimise outcomes and cost‐effectiveness (Hodgkins et al., 2013). In practice, this is predominantly based on subjective ratings (self‐report or based on parents’, teachers’ or clinicians’ impressions) that – although informative – may introduce bias in relation to the identification of clinically ‘meaningful’ or ‘informative’ changes in symptoms. Furthermore, individuals with ADHD (or their carers) might find it difficult to report changes accurately (Du Rietz et al., 2016), and there are discrepancies between self‐reports and objectively ascertained assessments (e.g. hyperactivity measured via actigraphy) (Lis et al., 2010). These challenges may potentially affect treatment adherence due to perceived lack of effect by the patients themselves, different opinions from different raters or different patient expectations as compared to clinicians (Cedergren, Östlund, Åsberg Johnels, Billstedt, & Johnson, 2022; Ramsay, 2017). Crucially, ADHD treatment response monitoring practices have not changed for decades and are often largely variable. Digital technology may help reduce this variability and may also support remote monitoring (Hall, Taylor, et al., 2016). However, as of today, there is no easily available objective measure that can be used for clinically monitoring ADHD treatment response (Michelini, Norman, Shaw, & Loo, 2022).

Finding biomarkers that are accurately associated with treatment response has indeed proven challenging. There is evidence that ADHD medication induces changes in neuro‐imaging, neuro‐cognitive and physiological measures, especially in the short term, but this varies across individuals and there is limited evidence in relation to long‐term changes (Faraone et al., 2021; Michelini et al., 2022; Parlatini et al., under review; Rubia et al., 2014). Furthermore, studies have found that changes detected in neuropsychological measures (e.g. reaction time, reaction time variability or performance accuracy) are only weakly associated with changes in ADHD symptoms or quality of life (Huang, Wang, & Chen, 2012; Inci Izmir, Ipci, & Ercan, 2022; Lee et al., 2022; Pievsky & McGrath, 2018), suggesting that cognitive tasks and symptom scales may capture partially distinct constructs (Kaiser et al., 2024). Similarly, pharmacological treatment for ADHD has been found to affect physiological measures, for instance heart rate variability (a measure of variation in cardiac activity over time which may reflect self‐regulation and is altered in ADHD; Bellato, Wiersema, & Groom, 2023), but the specific association with improvements in ADHD symptoms or quality of life is not clear (Buchhorn et al., 2012; Kim, Yang, & Lee, 2015). Importantly, most of these measures (especially neuroimaging or neurophysiological) are not routinely collected in day‐to‐day clinical practice, hence they are not suitable for monitoring ADHD treatment response.

A recent systematic review (Gustafsson & Hansen, 2023) investigated the possible use of the Quantitative Behaviour Test (QbTest; Qbtech Ltd, www.qbtech.com) for monitoring treatment response in ADHD. QbTest is a commercially available test involving activity monitoring via an infra‐red camera during a continuous performance task measuring sustained attention and response inhibition. QbTests’ summary scores are objective estimations of the three core symptoms of ADHD, i.e. attention, impulsivity and hyperactivity (Bellato, Hall et al., 2024; Hall, Selby, et al., 2016; Hall, Bellato, Kirk, & Hollis, 2023). The Food and Drug Administration (FDA) granted approval for the use of QbTest to aid clinical assessment/diagnosis of ADHD. A recently published guidance document from NICE (National Institute for Health and Care Excellence, 2024b) recommends that QbTest could be used as an option to help diagnose ADHD alongside standard clinical assessment in people aged 6–17 years. Moreover, NICE recommends that QbTest should be used only as a research tool to study ADHD treatment response monitoring, before more conclusive evidence is gathered (National Institute for Health and Care Excellence, 2024a). Conversely, in the U.S.A. the FDA approved the use of QbTest to aid treatment response monitoring in both adults and children (Dolgin, 2014). Nevertheless, both the FDA and NICE recommend that QbTest should not be used as a stand‐alone test, but its results should be interpreted in the context of a comprehensive clinical evaluation/assessment. This is in line with a recent study showing that QbTest does not discriminate between individuals with/without ADHD with sufficient accuracy to be used as a stand‐alone tool (Bellato, Hall, et al., 2024). Nevertheless, when following its intended use, QbTest may help speed up the diagnostic process and lead to more confident clinical decisions (Hollis et al., 2018; National Institute for Health and Care Excellence, 2024b).

Regarding the use of QbTest for ADHD treatment response monitoring, evidence is indeed still limited. The systematic review published by QbTech (Gustafsson & Hansen, 2023) identified 15 studies reporting QbTest scores before and after pharmacological treatment in children, young people and adults with ADHD. Overall, the authors observed improvements in QbTest performance in individuals with ADHD, following treatment with any medication, and concluded that ‘QbTest (..) can be used for monitoring of long‐term treatment of ADHD’. However, current evidence and limitations of the studies included in their systematic review may challenge such conclusive statement. Most studies (*n* = 12) assessed the effects of stimulants (e.g. methylphenidate or amphetamines) on QbTest parameters, two were on non‐stimulants (i.e. atomoxetine); however, one study on cannabidiol and studies with multiple or mixed medications were also included. Moreover, out of 15 studies, only six did have a placebo arm to compare medication‐related effects on QbTest parameters; potential expectation effects on QbTest performance cannot therefore be excluded and warrant further investigation. Additionally, half of the included studies (*n* = 7) reported the effects of a single dose of medication (e.g. after 2‐3 h), while the remaining studies (*n* = 8) reported long‐term effects but with different timelines (from 2 weeks up to 4 years), which introduce bias in the interpretation of findings. Gustafsson and Hansen (2023) reported positive effects of medication on QbTest parameters, but only weak association between changes in QbTest scores and ADHD symptoms (based on rating scales), and sometimes not in the long term. For instance, a study in adults with ADHD observed only small, although significant (all *r* < .33), correlations between QbTest scores and self‐rated symptom scales, both at baseline and after a month treatment (Bijlenga, Jasperse, Gehlhaar, & Sandra Kooij, 2015). These differences may be related to the fact that symptom scales rate the severity/frequency of complex behaviours in daily life, whilst QbTest assesses performance during a brief test in a controlled setting, thus they may reflect partially distinct constructs.

It would be interesting to understand how individuals with ADHD (particularly young people or those less inclined to begin pharmacological treatment) perceive QbTest results both before and after starting treatment. For instance, some may view changes in QbTest scores – potentially influenced by medication *effects* – as objective evidence of treatment *efficacy*. While this perception may promote initial adherence to treatment (especially for those less inclined to rely on self‐reports or feedback from parents, partners, or clinicians), it may present long‐term challenges if changes in QbTest scores do not align with improvements (or worsening) in core symptoms or other outcomes (e.g. mental health and global functioning). Given the scarcity of research in this area, we recommend further studies to better understand the potential benefits and challenges associated with using QbTest for ADHD treatment monitoring, as well as to explore the mechanisms underlying changes in ADHD symptoms and other domains in response to pharmacological treatments and combined interventions (i.e. integrating non‐pharmacological options with pharmacological treatments).

Understanding the perspectives of individuals with ADHD, parents/carers and healthcare professionals, about using digital technology (including QbTest) for treatment response monitoring, is crucial for informing future studies. For example, a feasibility randomised controlled trial (Hall et al., 2018; Williams et al., 2021) highlighted some challenges when including QbTest as an adjunct to routine practice, such as difficulties in carrying out follow‐ups in limited time periods. Nevertheless, QbTest was well accepted by clinicians and patients, who appreciated its objectivity (Williams et al., 2021). Another important question is whether QbTest captures what the young person/family prioritise as outcomes. This is equally true of traditional ADHD scales that prioritise core ADHD symptoms against other outcomes, highlighting the importance of using clinical measures that are generic and patient‐ or parent‐centred (Wolpert et al., 2017).

The currently available limited evidence should prompt future research to rigorously investigate the use of QbTest to monitor ADHD treatment response, possibly using a more rigorous randomised placebo‐controlled design to control for potential expectations and practice effects; considering different types of ADHD medications; and assessing the relationships between QbTest scores/parameters and self‐ or caregiver‐reported ADHD symptoms and quality of life. It would also be important to investigate the utility of QbTest for treatment monitoring both during titration and longer‐term, thus potentially helping clinicians reach a conclusion on treatment effectiveness, especially for those individuals that may find it harder to report on treatment‐related effects. As QbTest measures ADHD‐related difficulties against normative data, it may be potentially helpful to compare response to different medication dosages, aiding the titration process, but this needs further investigation (Hall et al., 2023). In parallel, more research is also needed to investigate the sensitivity of QbTest parameters in detecting changes that lead to clinical decisions about having reached dose optimisation. It may be helpful, for example, to investigate if and how much QbTest is sensitive to detect such changes as compared to self‐reported clinical measures. Finally, there is preliminary evidence that QbTest parameters measured pre‐treatment may enhance the accuracy of predictions of post‐treatment response, and this warrants further investigation (Parlatini et al., 2023, under review).

In conclusion, currently there is not sufficient evidence to recommend QbTest for monitoring response to pharmacological treatment for ADHD in clinical practice. However, further research is required to understand the acceptability, potential utility and cost‐effectiveness of QbTest in addition to clinical measures – as compared to clinical measures only – to track treatment‐related ADHD symptom changes during titration and longer‐term monitoring. If demonstrated to add value to the use of clinical measures only, the addition of QbTest might potentially guide more personalised and quicker treatment optimisation (e.g. changing treatment if first choice does not produce short‐term effects), which may be particularly helpful and resource saving in the context of growing demands for ADHD pharmacological treatment wi already overstretched clinical services.

**Key points**

- Accurately monitoring ADHD treatment response is challenging, as current approaches primarily rely on subjective ratings.
- There is not yet sufficient evidence to recommend QbTest for monitoring response to pharmacological treatment for ADHD in clinical practice.
- Rigorous clinical trials and research studies are needed to better understand the utility of QbTest for monitoring ADHD treatment response.

## Data Availability

No original or new data have been reported or discussed in the commentary.
